# Supplementary material for: Global burden and trends of tracheal, bronchus, and lung cancer attributed to occupational exposure to polycyclic aromatic hydrocarbons in regions with different sociodemographic index, 1990–2021
Source: PLoS One. 2026 Feb 12;21(2):e0342250. doi: 10.1371/journal.pone.0342250 (PMC12900364; doi:10.1371/journal.pone.0342250)
Supplement: S1 Table — (PDF) [file pone.0342250.s001.pdf]

S1 Table. Burden and trends of TBL cancer attributed to occupational exposure to PAHs in regions with different SDI by sex, 1990-2021.

Population	Region	ASDR (per 100,000) and 95% UI				Age-standardized DALYs (per 100,000) and 95% UI		
		1990 year	2021 year	EAPC (%)		1990 year	2021 year	EAPC (%)
Female	Global	0.03 (0.02, 0.03)	0.05 (0.04, 0.06)	1.71 (1.59, 1.82)		0.81 (0.64, 0.99)	1.35 (1.05, 1.74)	1.46 (1.34, 1.58)
	Low SDI	0.02 (0.02, 0.03)	0.02 (0.02, 0.03)	1.30 (1.20, 1.41)		0.65 (0.53, 0.78)	0.69 (0.57, 0.84)	1.21 (1.10, 1.33)
	Low-middle SDI	0.03 (0.02, 0.04)	0.07 (0.05, 0.10)	2.15 (2.07, 2.23)		0.99 (0.76, 1.23)	2.14 (1.56, 2.87)	2.04 (1.96, 2.12)
	Middle SDI	0.00 (0.00, 0.01)	0.01 (0.01, 0.01)	1.33 (1.24, 1.41)		0.15 (0.11, 0.20)	0.23 (0.17, 0.30)	1.05 (0.97, 1.13)
	High-middle SDI	0.01 (0.01, 0.01)	0.02 (0.01, 0.02)	2.63 (2.40, 2.87)		0.26 (0.20, 0.32)	0.48 (0.37, 0.59)	2.29 (2.04, 2.54)
	High SDI	0.04 (0.03, 0.05)	0.07 (0.05, 0.09)	0.39 (0.27, 0.52)		1.27 (0.99, 1.58)	1.90 (1.47, 2.48)	0.21 (0.07, 0.36)
Male	Global	0.08 (0.06, 0.10)	0.09 (0.07, 0.12)	0.36 (0.28, 0.44)		2.53 (1.98, 3.15)	2.64 (2.01, 3.38)	0.16 (0.08, 0.24)
	Low SDI	0.06 (0.05, 0.08)	0.04 (0.03, 0.05)	0.30 (0.23, 0.37)		1.89 (1.51, 2.31)	1.16 (0.94, 1.42)	0.24 (0.17, 0.31)
	Low-middle SDI	0.13 (0.10, 0.16)	0.14 (0.10, 0.19)	1.17 (1.09, 1.24)		3.93 (3.06, 4.98)	4.08 (2.99, 5.53)	1.07 (1.00, 1.14)
	Middle SDI	0.02 (0.01, 0.03)	0.02 (0.02, 0.03)	0.69 (0.58, 0.79)		0.62 (0.46, 0.90)	0.70 (0.52, 0.92)	0.46 (0.36, 0.57)
	High-middle SDI	0.03 (0.03, 0.05)	0.05 (0.04, 0.06)	0.37 (0.25, 0.49)		1.10 (0.85, 1.48)	1.50 (1.19, 1.82)	0.10 (-0.02, 0.21)
	High SDI	0.10 (0.08, 0.13)	0.12 (0.09, 0.16)	-1.37 (-1.42, -1.32)		3.13 (2.42, 3.97)	3.56 (2.63, 4.68)	-1.56 (-1.61, -1.51)

Note: ASDR, age-standardized death rate; DALYs, disability adjusted life-years; EPAC, estimated annual percentage change; SDI, socio-demographic index; UI, uncertainty interval.
