# Supplementary figures and images for: Global burden and trends of tracheal, bronchus, and lung cancer attributed to occupational exposure to polycyclic aromatic hydrocarbons in regions with different sociodemographic index, 1990–2021
Source: PLoS One. 2026 Feb 12;21(2):e0342250. doi: 10.1371/journal.pone.0342250 (PMC12900364; doi:10.1371/journal.pone.0342250)

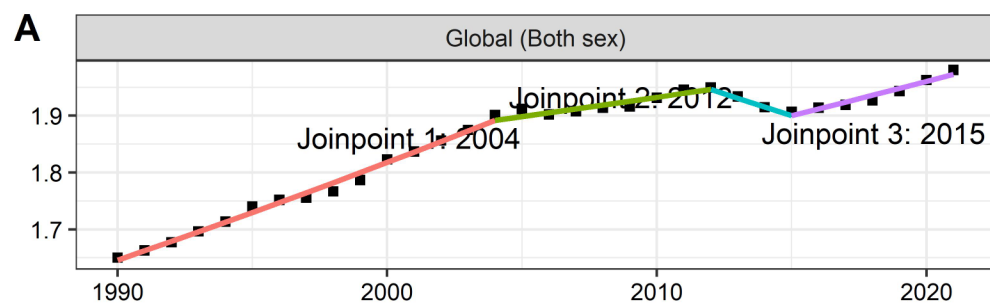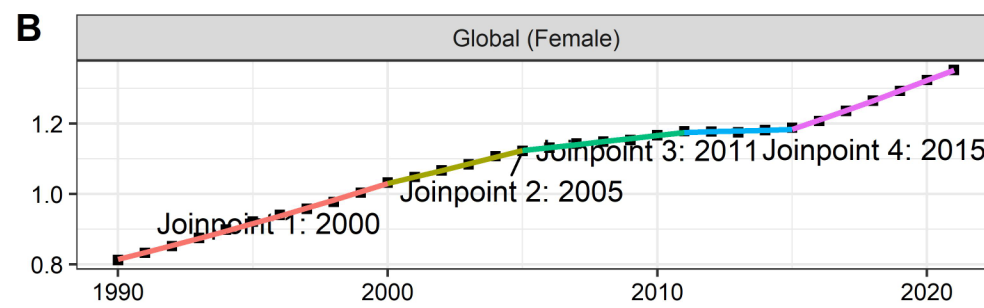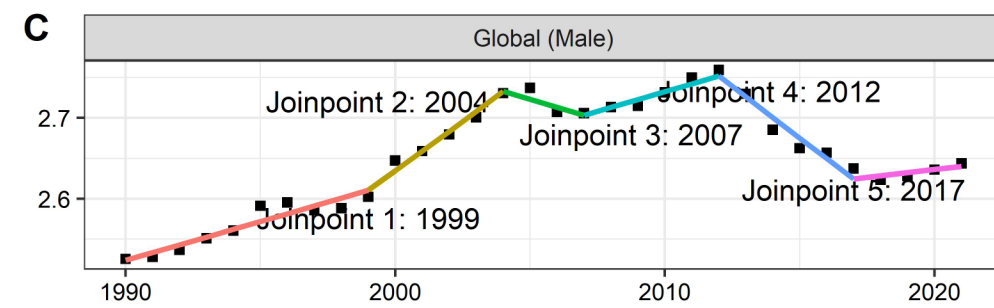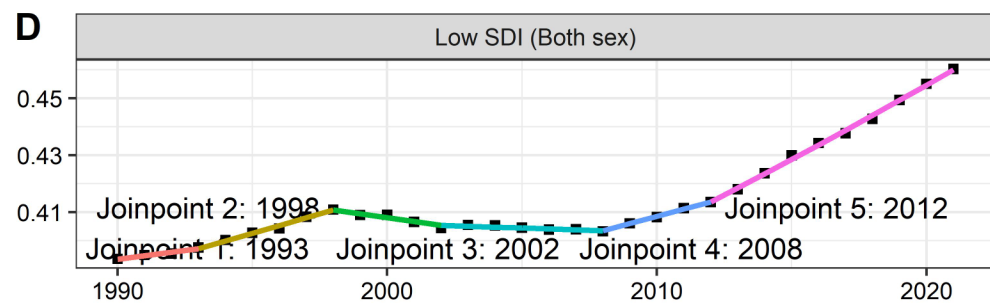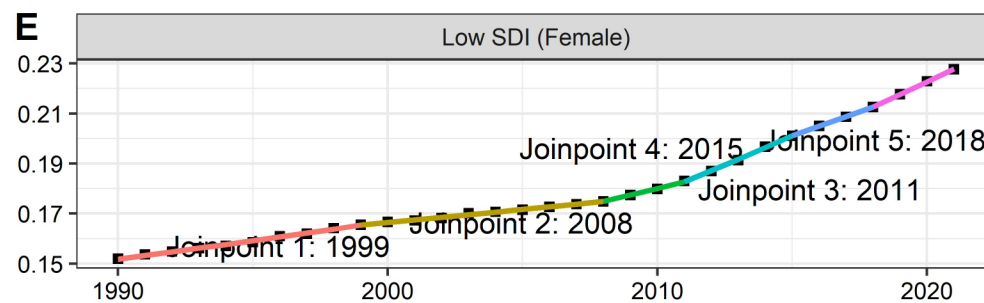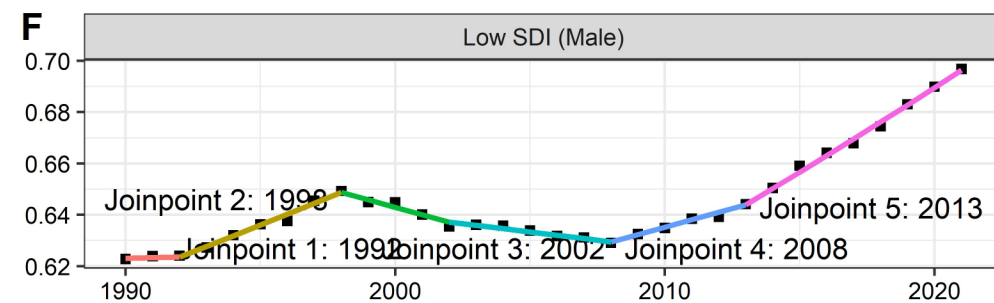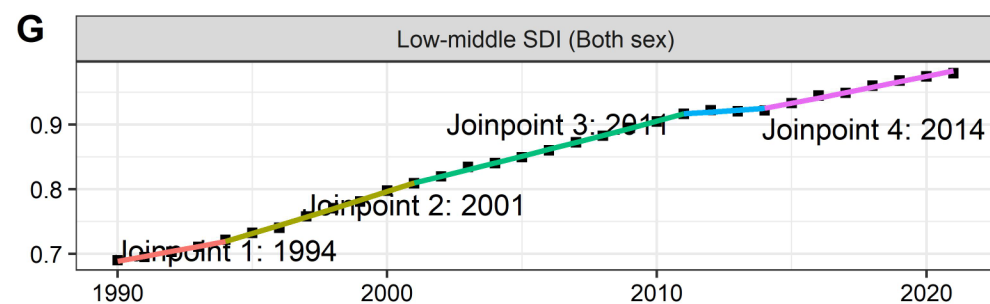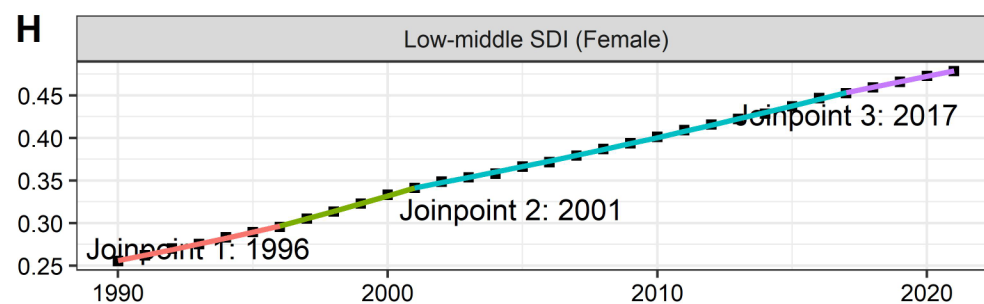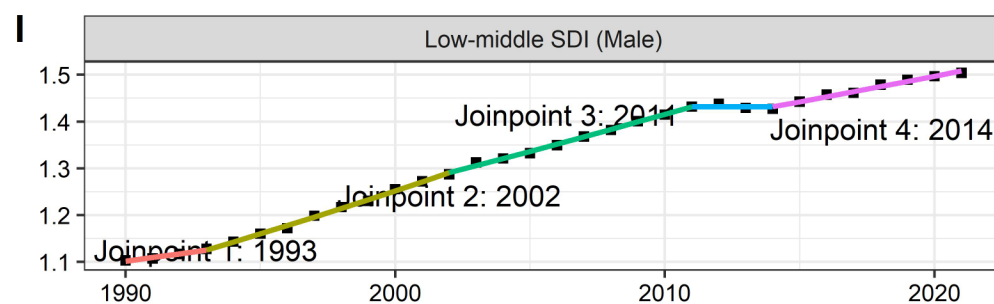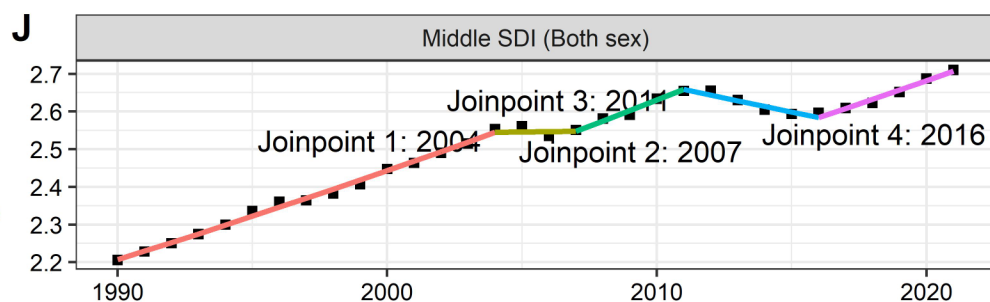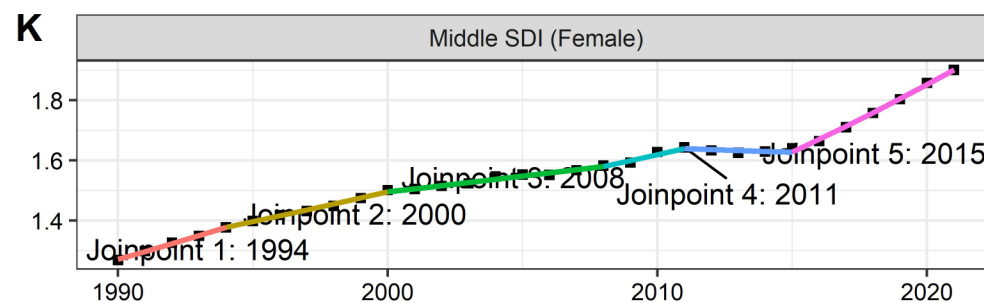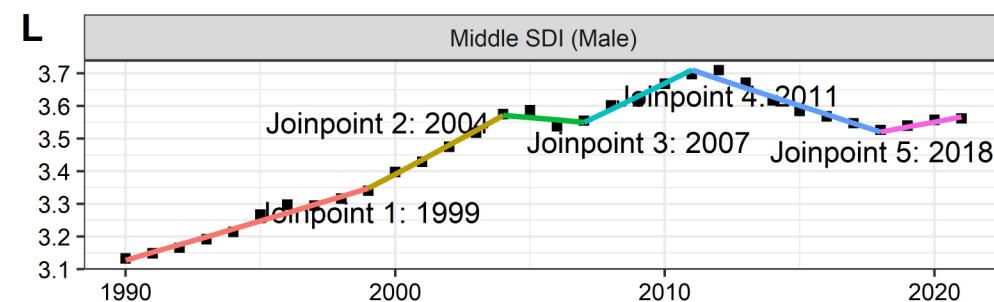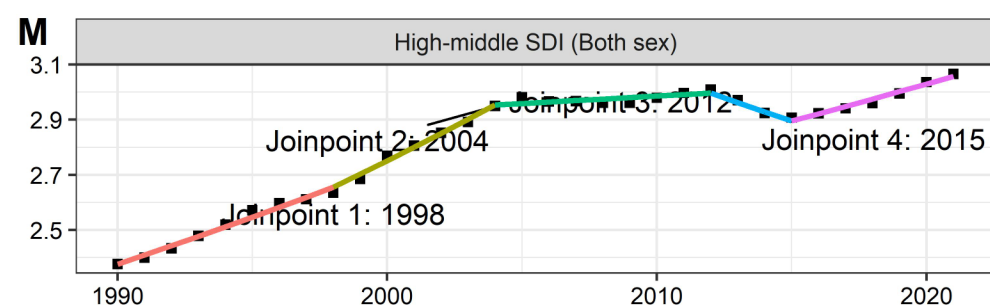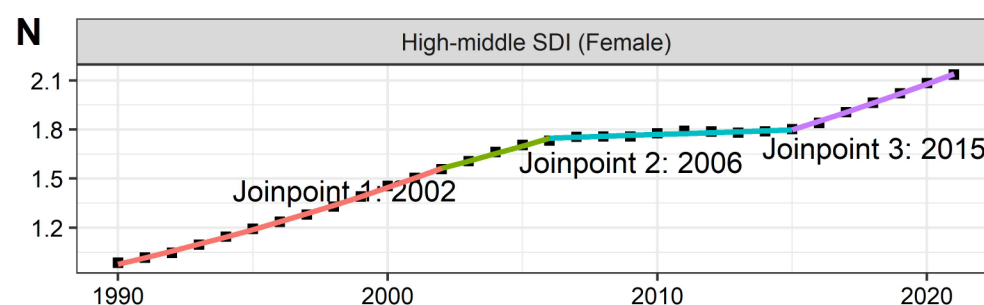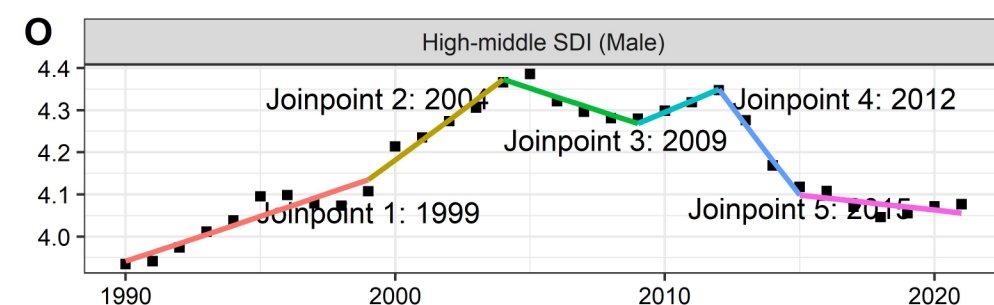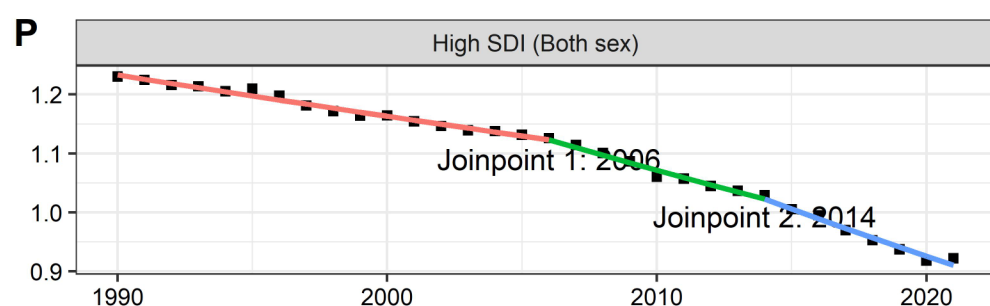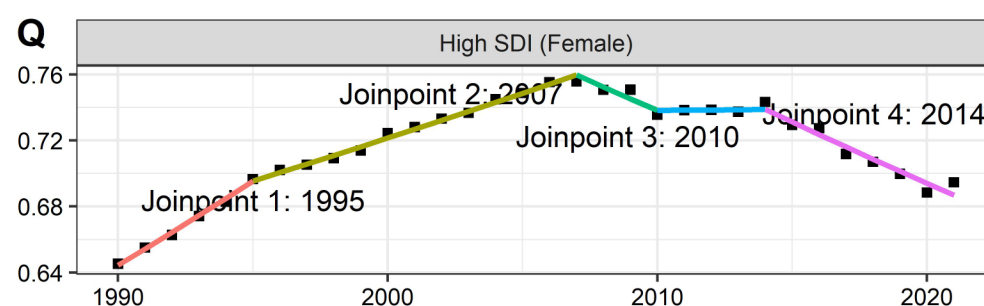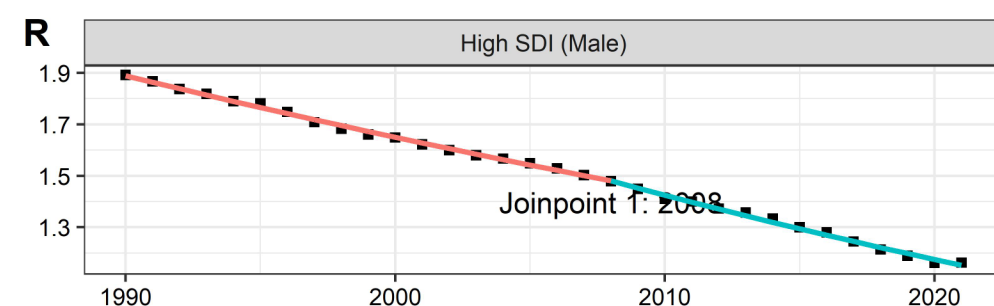

Supplement: S1 Fig — (A-C) Global; (D-F) Low SDI; (G-I) Low-middle SDI; (J-L) Middle SDI; (M-O) High-middle SDI; (P-R) High SDI. Note: DALYs, disability adjusted life-years; SDI, socio-demographic index. (PDF) [file pone.0342250.s002.pdf]

Age-standardized DALYs rate

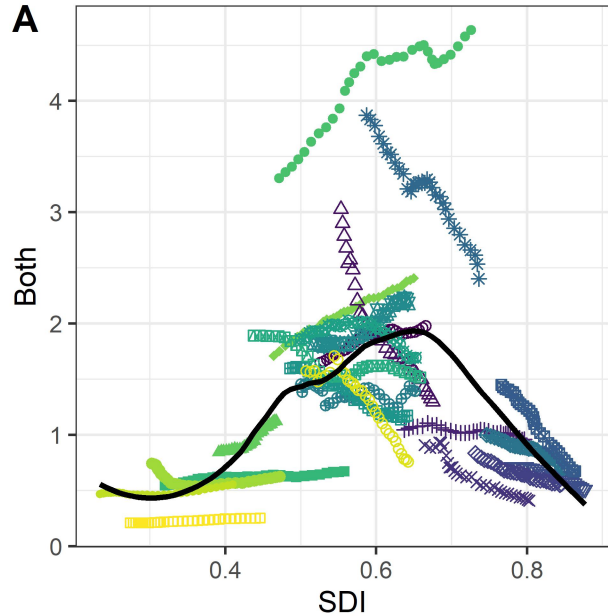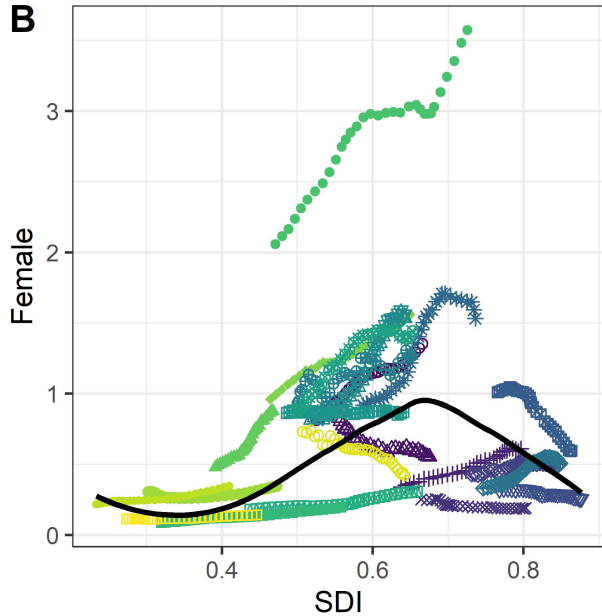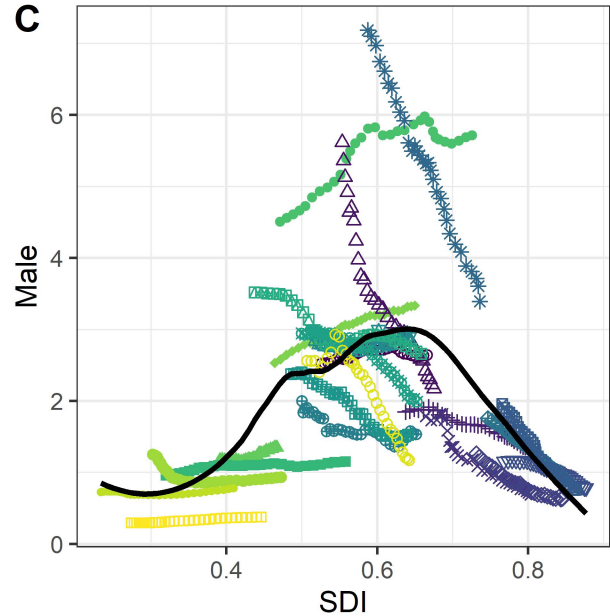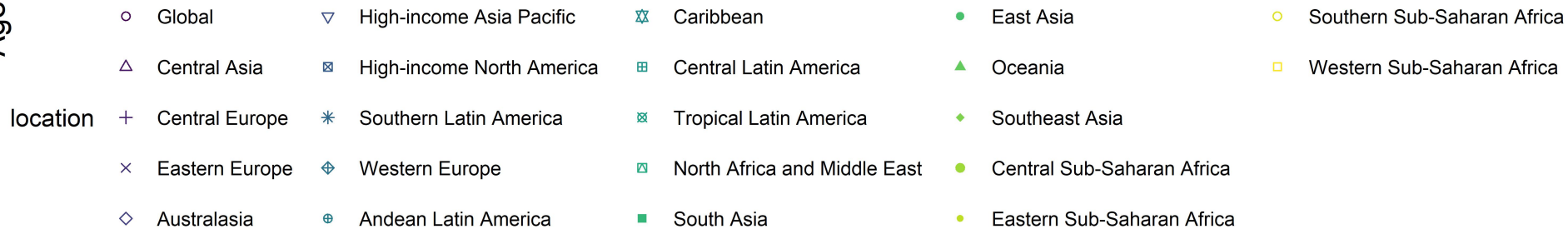

Supplement: S4 Fig — (A) Both; (B) Female; (C) Male. Note: DALYs, disability adjusted life-years; SDI, socio-demographic index. (PDF) [file pone.0342250.s005.pdf]

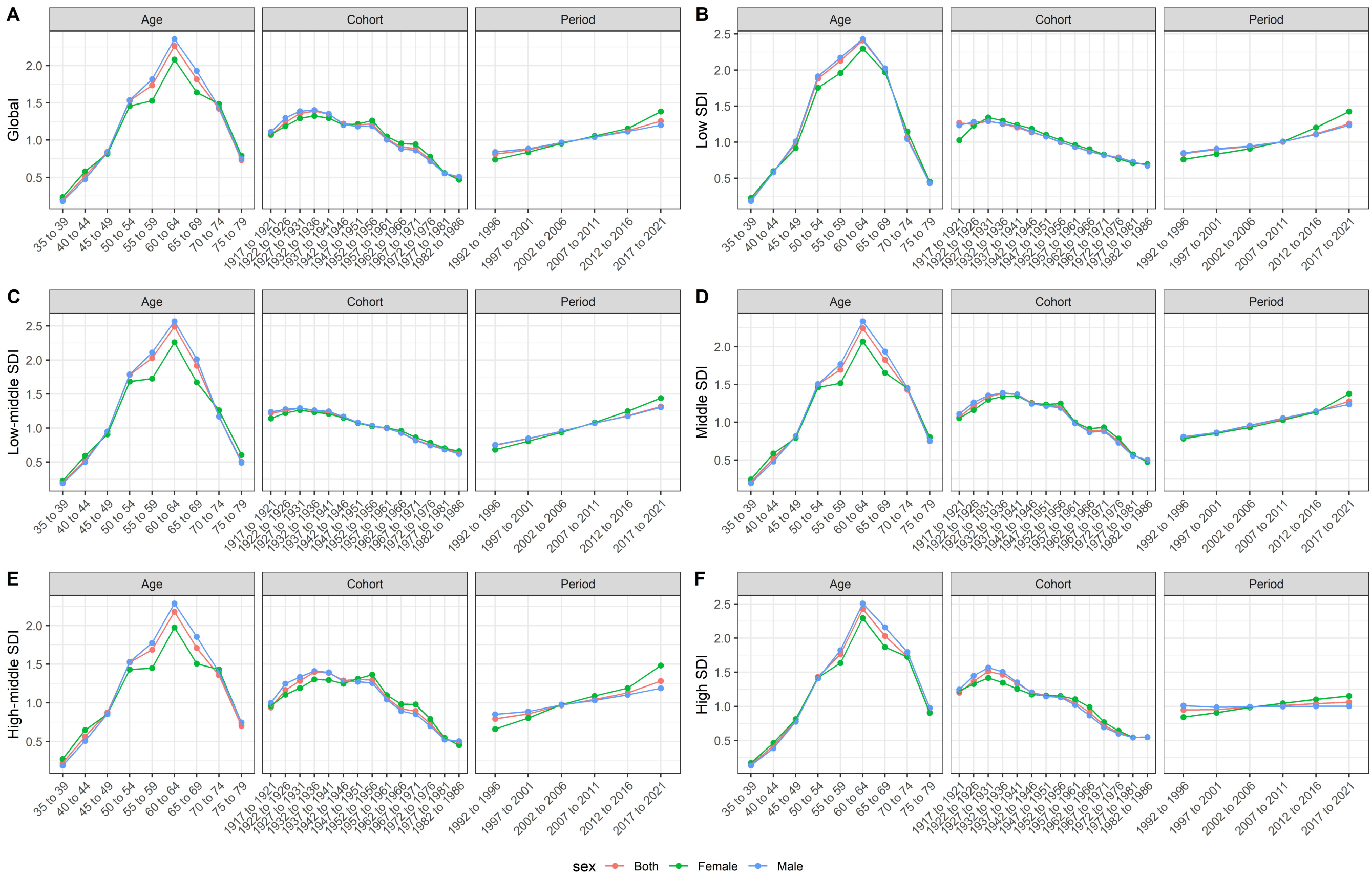

Supplement: S5 Fig — (A) Global; (B) Low SDI; (C) Low-middle SDI; (D) Middle SDI; (E) High-middle SDI; (F) High SDI. Note: DALYs, disability adjusted life-years; SDI, socio-demographic index. (PDF) [file pone.0342250.s006.pdf]
