# Supplementary material for: Global burden and trends of tracheal, bronchus, and lung cancer attributed to occupational exposure to polycyclic aromatic hydrocarbons in regions with different sociodemographic index, 1990–2021
Source: PLoS One. 2026 Feb 12;21(2):e0342250. doi: 10.1371/journal.pone.0342250 (PMC12900364; doi:10.1371/journal.pone.0342250)
Supplement: S2 Fig — (A-C) 1990; (D-F) 2021. Note: DALYs, disability adjusted life-years; SDI, socio-demographic index. (PDF) [file pone.0342250.s003.pdf]

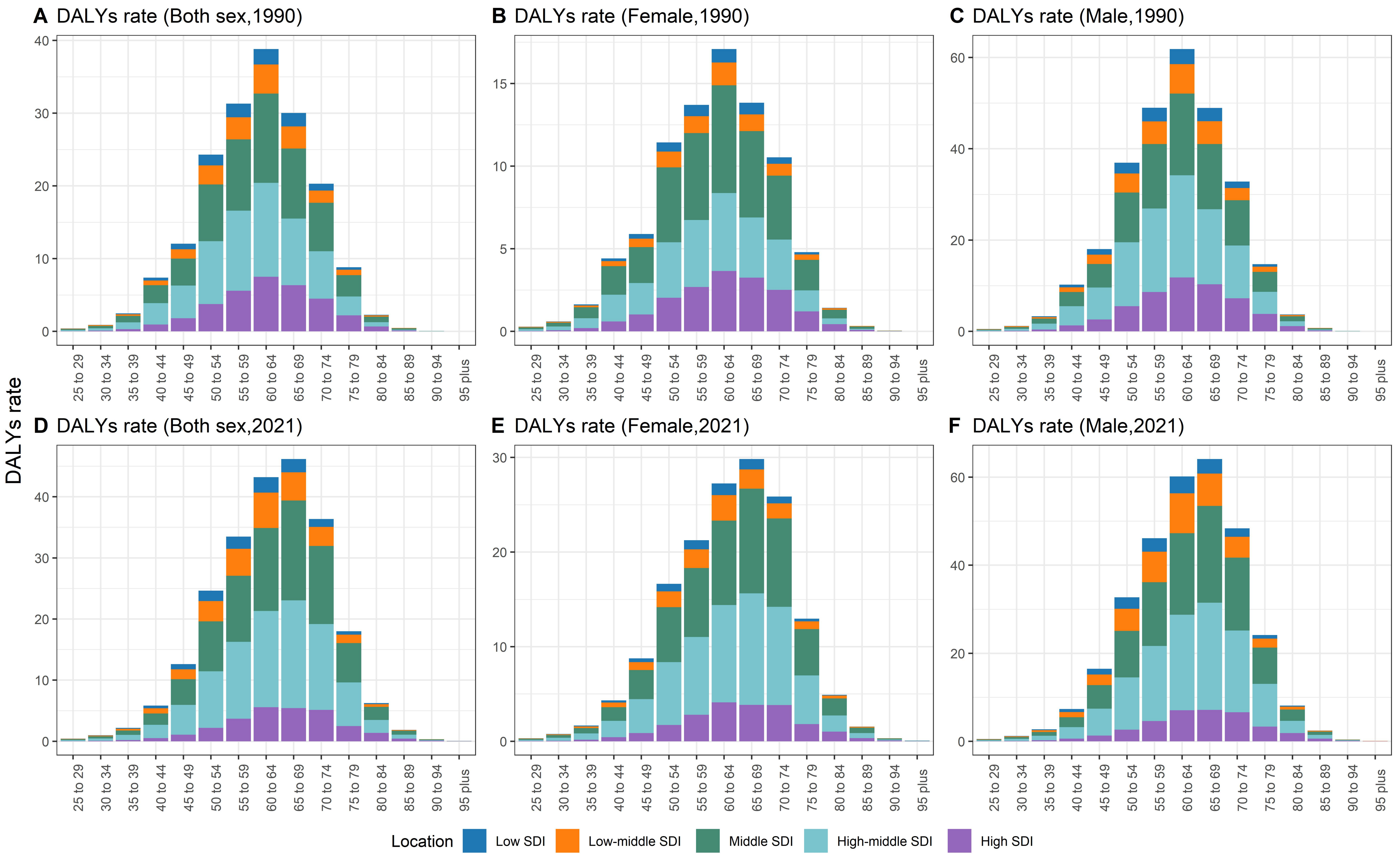

A DALYs rate (Both sex,1990)
B DALYs rate (Female,1990)
C DALYs rate (Male,1990)
D DALYs rate (Both sex,2021)
E DALYs rate (Female,2021)
F DALYs rate (Male,2021)
DALYs rate
25 to 29
30 to 34
35 to 39
40 to 44
45 to 49
50 to 54
55 to 59
60 to 64
65 to 69
70 to 74
75 to 79
80 to 84
85 to 89
90 to 94
95 plus
Location
Low SDI
Low-middle SDI
Middle SDI
High-middle SDI
High SDI
