# Supplementary material for: Global burden and trends of tracheal, bronchus, and lung cancer attributed to occupational exposure to polycyclic aromatic hydrocarbons in regions with different sociodemographic index, 1990–2021
Source: PLoS One. 2026 Feb 12;21(2):e0342250. doi: 10.1371/journal.pone.0342250 (PMC12900364; doi:10.1371/journal.pone.0342250)
Supplement: S3 Fig — (A) Both; (B) Female; (C) Male. Note: DALYs, disability adjusted life-years; EPAC, estimated annual percentage change; SDI, socio-demographic index. (PDF) [file pone.0342250.s004.pdf]

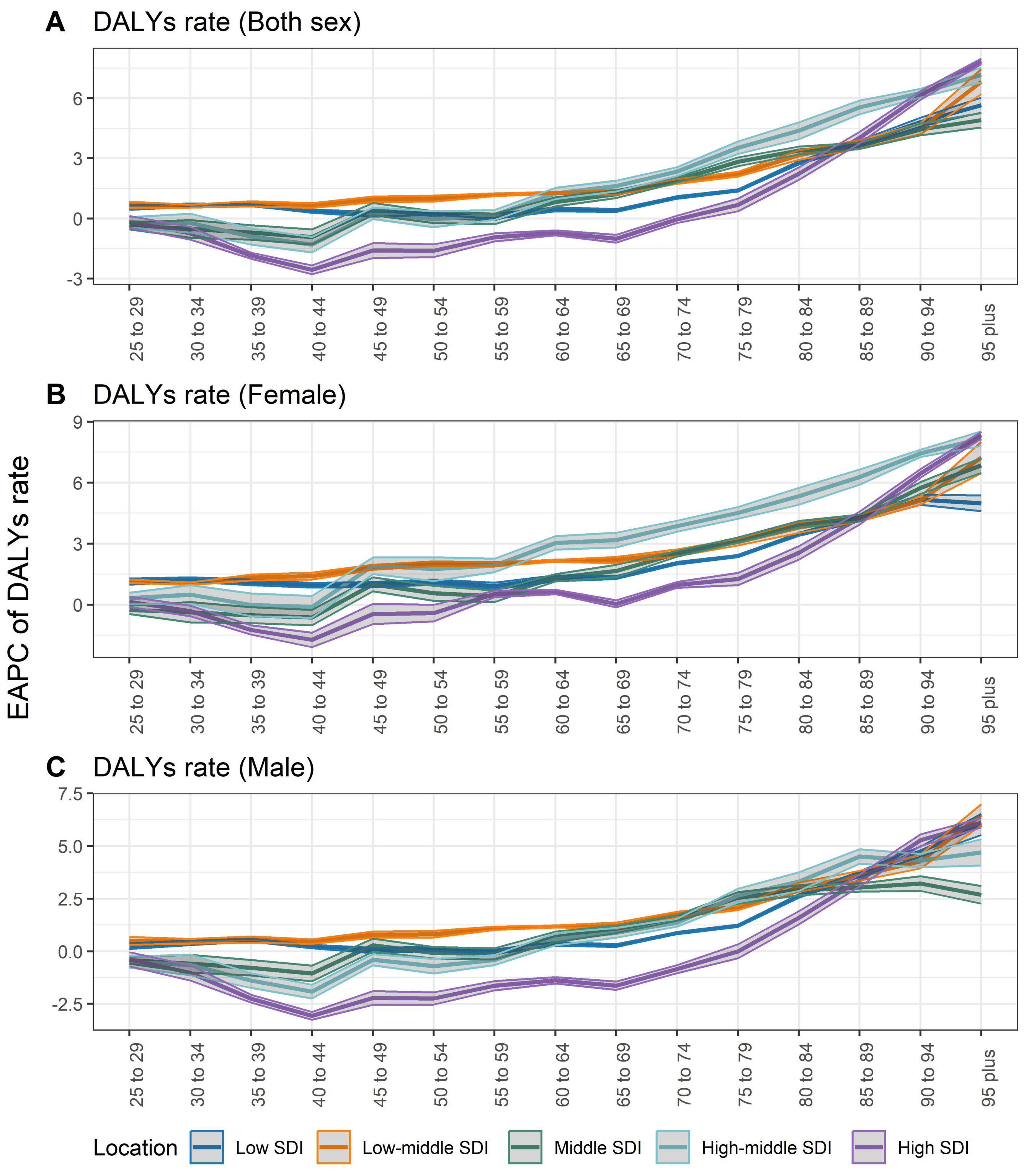
A
DALYs rate (Both sex)
B
DALYs rate (Female)
C
DALYs rate (Male)
EAPC of DALYs rate
6
3
0
-3
9
7.5
5.0
2.5
0.0
-2.5
25 to 29
30 to 34
35 to 39
40 to 44
45 to 49
50 to 54
55 to 59
60 to 64
65 to 69
70 to 74
75 to 79
80 to 84
85 to 89
90 to 94
95 plus
Location
Low SDI
Low-middle SDI
Middle SDI
High-middle SDI
High SDI
