# Supplementary material for: Global burden and trends of tracheal, bronchus, and lung cancer attributed to occupational exposure to polycyclic aromatic hydrocarbons in regions with different sociodemographic index, 1990–2021
Source: PLoS One. 2026 Feb 12;21(2):e0342250. doi: 10.1371/journal.pone.0342250 (PMC12900364; doi:10.1371/journal.pone.0342250)
Supplement: Attachment — Submitted filename: Response letter.docx [file pone.0342250.s009.docx]

PONE-D-25-19840

Global burden and trends of tracheal, bronchus, and lung cancer attributed to occupational exposure to polycyclic aromatic hydrocarbons in regions with different sociodemographic index, 1990-2021

PLOS ONE

**Response letter**

Dear Dr. Oghenetega,

We appreciate your insightful feedback. We have carefully revised the manuscript in accordance with recommendations and believe that these comments have significantly enhanced the manuscript. A point-by-point response to the comments is attached along with the revised manuscript.

We appreciate your guidance and look forward to your response.

Sincerely,

Xiaowei Xie

Department of Thoracic Surgery, The First Hospital of Putian City, Putian, 351100, China

Email: xxw_biolab@163.com

***Journal Requirements***

*1. When submitting your revision, we need you to address these additional requirements.*

*Please ensure that your manuscript meets PLOS ONE's style requirements, including those for file naming. The PLOS ONE style templates can be found at*

*https://journals.plos.org/plosone/s/file?id=wjVg/PLOSOne_formatting_sample_main_body.pdf and*

[*https://journals.plos.org/plosone/s/file?id=ba62/PLOSOne_formatting_sample_title_authors_affiliations.pdf*](https://journals.plos.org/plosone/s/file?id=ba62/PLOSOne_formatting_sample_title_authors_affiliations.pdf)

Response: Done. Thank you.

*2. When completing the data availability statement of the submission form, you indicated that you will make your data available on acceptance. We strongly recommend all authors decide on a data sharing plan before acceptance, as the process can be lengthy and hold up publication timelines. Please note that, though access restrictions are acceptable now, your entire data will need to be made freely accessible if your manuscript is accepted for publication. This policy applies to all data except where public deposition would breach compliance with the protocol approved by your research ethics board. If you are unable to adhere to our open data policy, please kindly revise your statement to explain your reasoning and we will seek the editor's input on an exemption. Please be assured that, once you have provided your new statement, the assessment of your exemption will not hold up the peer review process.*

Response: Thank you for this comment. All the data used in this study are publicly available, and we have included this in the manuscript: The GBD 2021 data used in this study are publicly available online at https://www.healthdata.org/.

*3. PLOS requires an ORCID iD for the corresponding author in Editorial Manager on papers submitted after December 6th, 2016. Please ensure that you have an ORCID iD and that it is validated in Editorial Manager. To do this, go to ‘Update my Information’ (in the upper left-hand corner of the main menu), and click on the Fetch/Validate link next to the ORCID field. This will take you to the ORCID site and allow you to create a new iD or authenticate a pre-existing iD in Editorial Manager.*

Response: Done. Thank you.

*4. We note that Figure 1 in your submission contain map images which may be copyrighted. All PLOS content is published under the Creative Commons Attribution License (CC BY 4.0), which means that the manuscript, images, and Supporting Information files will be freely available online, and any third party is permitted to access, download, copy, distribute, and use these materials in any way, even commercially, with proper attribution. For these reasons, we cannot publish previously copyrighted maps or satellite images created using proprietary data, such as Google software (Google Maps, Street View, and Earth). For more information, see our copyright guidelines: http://journals.plos.org/plosone/s/licenses-and-copyright.*

*We require you to either present written permission from the copyright holder to publish these figures specifically under the CC BY 4.0 license, or remove the figures from your submission:*

*a. You may seek permission from the original copyright holder of Figure 1 to publish the content specifically under the CC BY 4.0 license.*

*We recommend that you contact the original copyright holder with the Content Permission Form (http://journals.plos.org/plosone/s/file?id=7c09/content-permission-form.pdf) and the following text:*

*“I request permission for the open-access journal PLOS ONE to publish XXX under the Creative Commons Attribution License (CCAL) CC BY 4.0 (http://creativecommons.org/licenses/by/4.0/). Please be aware that this license allows unrestricted use and distribution, even commercially, by third parties. Please reply and provide explicit written permission to publish XXX under a CC BY license and complete the attached form.”*

*Please upload the completed Content Permission Form or other proof of granted permissions as an "Other" file with your submission.*

*In the figure caption of the copyrighted figure, please include the following text: “Reprinted from [ref] under a CC BY license, with permission from [name of publisher], original copyright [original copyright year].”*

*b. If you are unable to obtain permission from the original copyright holder to publish these figures under the CC BY 4.0 license or if the copyright holder’s requirements are incompatible with the CC BY 4.0 license, please either i) remove the figure or ii) supply a replacement figure that complies with the CC BY 4.0 license. Please check copyright information on all replacement figures and update the figure caption with source information. If applicable, please specify in the figure caption text when a figure is similar but not identical to the original image and is therefore for illustrative purposes only.*

*The following resources for replacing copyrighted map figures may be helpful:*

*USGS National Map Viewer (public domain): http://viewer.nationalmap.gov/viewer/*

*The Gateway to Astronaut Photography of Earth (public domain): http://eol.jsc.nasa.gov/sseop/clickmap/*

*Maps at the CIA (public domain): https://www.cia.gov/library/publications/the-world-factbook/index.html and https://www.cia.gov/library/publications/cia-maps-publications/index.html*

*NASA Earth Observatory (public domain): http://earthobservatory.nasa.gov/*

*Landsat: http://landsat.visibleearth.nasa.gov/*

*USGS EROS (Earth Resources Observatory and Science (EROS) Center) (public domain): http://eros.usgs.gov/#*

*Natural Earth (public domain): http://www.naturalearthdata.com/*

Response: Done. Thank you for this kind remind. In our study, world map shapefile was obtained from the Resources and Environmental Science Data Platform (RESDC), which is in the public domain and publicly accessible at <https://www.resdc.cn/>. We have added this for Figure 1.

5. Please include captions for your Supporting Information files at the end of your manuscript, and update any in-text citations to match accordingly. Please see our Supporting Information guidelines for more information: <http://journals.plos.org/plosone/s/supporting-information>.

Response: Done. Thank you.

*6. If the reviewer comments include a recommendation to cite specific previously published works, please review and evaluate these publications to determine whether they are relevant and should be cited. There is no requirement to cite these works unless the editor has indicated otherwise.*

Response: No publications recommended. Thank you for this kind remind.

***Editor***

Reviewers commend the study but request clearer model assumptions and sensitivity analyses for LMIC data, fuller explanation of GBD exposure attribution, discussion of co-exposure confounding, mechanistic insight into the SDI-disease burden pattern, control of smoking confounding, and rationale for sex differences. They also urge stronger policy and intervention recommendations, especially for low- and middle-SDI regions. These revisions will improve clarity and relevance.

Response: Thank you for your thoughtful comments and considerations. We have improved our manuscript following your and the reviewers’ comments. The detailed responses to the reviewers are provided in this letter.

***Reviewer 1***

*1. The assumptions for the model may be clearly delineated in the methods.* *Also, the* *quality of the data from LMICs may be poor, hence sensitivity analyses may be conducted to unravel effect of the assumptions made*

Response: Thank you for this important comment. We agree that a more detailed explanation of the modeling assumptions is crucial. As our study is a secondary analysis of the Global Burden of Disease (GBD) data, the core assumptions and limitations are inherent to the GBD framework itself, and we could not directly calculate the estimates after excluding the original data from LMICs. We have added more content on assumptions for the model and overall quality of the data from LMICs.

In addition, the GBD framework utilizes models like spatiotemporal Gaussian process regression (ST-GPR) to pool this heterogeneous data. These models are crucial for controlling and adjusting for bias and for generating estimates in locations or time periods where direct data may be limited [1]. Nevertheless, it is true that one inherent limitation of the GBD study is the relatively poor data in LMICs.

We have added related content as:

**2.4 Methodology to calculate attributable burden in the GBD framework**

To quantify the disease burden attributable to a specific risk factor, the GBD study used a validated comparative risk assessment (CRA) framework [1]. In brief, the first step is quantifying the relative risks (RRs) of the health outcome (TBL cancer) as a function of exposure to the risk factor (occupational PAHs). This was done using a meta-regression in a “burden of proof” approach, which synthesizes data from systematic reviews. Second, exposure data are projected to the global scale from various sources using Bayesian statistical models, specifically spatiotemporal Gaussian process regression. Third, by integrating projected exposure levels, attributable burden could be calculated to quantify the proportional change in TBL cancer burden that would occur if occupational PAH exposure was reduced to the TMREL. Fourth, the GBD framework accounts for the joint effects of risk factors by assuming that RRs are multiplicative. For risk factors without mediating pathways, such as occupational PAH exposure and confounding factors (e.g., smoking and other occupational carcinogens), their independent contributions were calibrated to avoid overestimation of joint effects [1]. More detailed descriptions on the statistical methods, input data, and exposure-response functions are publicly accessible online (https://ghdx.healthdata.org/record/ihme-data/gbd-2021-burden-by-risk-1990-2021). Finally, all related estimates were recorded in the GBD 2021 result tools (<https://vizhub.healthdata.org/gbd-results/>). **(Method Section)**

First, a key limitation of our study is that the GBD framework is subjected to relatively incomplete and heterogeneous data from LMICs. While the use of spatiotemporal Gaussian process regression helps mitigate data gaps by integrating multiple sources and adjusting for bias, substantial underreporting of occupational exposures and TBL cancer cases in LMICs may lead to underestimations of the true burden [1]. **(Discussion Section)**

*2. The manuscript would benefit from more detail on the* *methodology used by GBD to assign causality between occupational PAH exposure and TBL cancer.*

Response: We agree and appreciate your suggestion, and have added related content as: **2.4 Methodology to calculate attributable burden in the GBD framework**

To quantify the disease burden attributable to a specific risk factor, the GBD study used a validated comparative risk assessment (CRA) framework [1]. In brief, the first step is quantifying the relative risks (RRs) of the health outcome (TBL cancer) as a function of exposure to the risk factor (occupational PAHs). This was done using a meta-regression in a “burden of proof” approach, which synthesizes data from systematic reviews. Second, exposure data are projected to the global scale from various sources using Bayesian statistical models, specifically spatiotemporal Gaussian process regression. Third, by integrating projected exposure levels, attributable burden could be calculated to quantify the proportional change in TBL cancer burden that would occur if occupational PAH exposure was reduced to the TMREL. Fourth, the GBD framework accounts for the joint effects of risk factors by assuming that RRs are multiplicative. For risk factors without mediating pathways, such as occupational PAH exposure and confounding factors (e.g., smoking and other occupational carcinogens), their independent contributions were calibrated to avoid overestimation of joint effects [1]. More detailed descriptions on the statistical methods, input data, and exposure-response functions are publicly accessible online (https://ghdx.healthdata.org/record/ihme-data/gbd-2021-burden-by-risk-1990-2021). Finally, all related estimates were recorded in the GBD 2021 result tools (<https://vizhub.healthdata.org/gbd-results/>). **(Method Section)**

*3. Consider briefly discussing confounding by other occupational exposures (e.g.,* *asbestos, silica), which are common co-exposures and may overlap in industrial settings.*

Response: Thank you for this comment. We have added related content: Previous studies examining the health burden of TBL cancer have predominantly focused on outdoor air pollution (e.g., emissions from vehicles and industrial activities) and other occupational hazards (e.g., asbestos, silica) [2-5]. **(Introduction Section)** & Last, while the GBD framework used techniques to adjust major risk factors as independent contributors, it could not fully account for potential interactions between occupational PAHs and other exposures, such as outdoor air pollution and co-occurring occupational carcinogens (e.g., asbestos, silica). **(Discussion Section)**

*4. The finding of an inverted U-shaped relationship between SDI and disease burden is interesting but underexplored. The authors should provide greater mechanistic or contextual explanation—e.g., linking it to patterns in industrialization, occupational regulation, and latency of cancer onset.*

Response: We agree and appreciate your comments. We have added related discussions according to your comments: First, older populations in these regions may have experienced prolonged exposure to occupational PAHs due to delayed implementation or absence of strict workplace safety regulations [6-8]. & In low to middle SDI regions, rapid industrialization particularly in sectors with high PAH exposure risks, such as manufacturing (e.g., aluminum production, coal tar distillation), construction, and informal waste management drives elevated occupational exposure [9, 10]. Meanwhile, inadequate protective measures such as poor ventilation and limited personal protective equipment may also aggravate related disease burden. This trend is particularly pronounced in regions such as East Asia, where accelerated industrialization in the late 20th century coincided with delayed adoption of workplace safety standards [11, 12]. The less apparent decline in females in high SDI regions also suggests potential gender-related disparities in occupational exposure patterns [12]. As regions transition to high SDI, advancements in occupational health policies, automation, and cleaner technologies reduce direct PAH exposure. Stricter regulatory frameworks combined with shifts toward service-oriented economies may also mitigate the TBL cancer burden attributed to occupational exposure to PAHs [6, 8]. **(Discussion Section)**

*5.The paper would benefit from a stronger policy or practical intervention focus, especially in the conclusion. For example, what are the implications for workplace safety standards, surveillance, or global occupational health policy?*

Response: Thank you for this important comment. We have related content in Discussion as: To reduce the global burden of TBL cancer associated with occupational PAH exposure, policymakers should prioritize interventions that align with the socio-economic and industrial contexts of regions at different SDI levels. Particularly, in low- and middle-SDI regions, where industrialization often outpaces occupational safety infrastructure, governments should enforce stricter workplace exposure limits for PAHs and the use of personal protective equipment. Globally, harmonizing occupational safety standards through frameworks such as the ILO conventions and promoting technology transfer from high- to low-resource settings could mitigate disparities. Additionally, integrating gender-specific protections such as targeted safety protocols in female-dominated occupations would also be potentially helpful to mitigate related health burden. **(Discussion Section)**

***Reviewer 2***

This study provides a timely and comprehensive analysis of the global burden of tracheal, bronchus, and lung (TBL) cancer attributable to occupational polycyclic aromatic hydrocarbon (PAH) exposure, highlighting disparities across socio-demographic index (SDI) regions from 1990 to 2021. Its strengths include rigorous use of Global Burden of Disease (GBD) data, detailed age-period-cohort analyses, and insightful stratification by SDI, revealing concerning trends in low- and middle-income regions. However, limitations such as reliance on modeled estimates, potential confounding by smoking, and lack of occupational exposure details (e.g., duration, industry-specific risks) warrant clarification. The findings underscore the need for targeted workplace interventions but would benefit from deeper discussion on mechanistic pathways and policy implications to strengthen impact. Overall, the study advances understanding of occupational cancer disparities but requires minor methodological refinements and expanded contextualization for broader relevance.

*1. The study relies on GBD estimates. Could you elaborate on how occupational PAH exposure was specifically measured and attributed to TBL cancer cases in the GBD data? What validation exists for these exposure estimates?*

Response: We appreciate you for this important comment. We have added more information on the details related data we used in the **Method Section**.

**2.2 Occupational exposure to PAHs and TBL cancer**

Data on occupational exposure to PAHs were derived from the GBD study (https://vizhub.healthdata.org/gbd-compare/), with detailed information on data sources and methodology publicly available online (<https://www.healthdata.org/gbd/methods-appendices-2021/occupational-risk-factors>).

In brief, occupational exposure to PAHs was quantified within the GBD framework using a multi-step process integrating economic activity classifications, occupation distributions, and exposure risk levels [13]. The input data were primarily sourced from the International Labor Organization (ILO). Occupational PAH exposure levels (high/low) were categorized based on the 17 International Standard Industrial Classification (ISIC) economic activities and 9 International Standard Classification of Occupations (ISCO) occupational categories. According to the GBD framework, the Theoretical Minimum Risk Exposure Level (TMREL) of occupational exposure to PAHs was set to zero (no thresholds). High-exposure industries (e.g., coal mining, asphalt production) were assigned elevated exposure rates using the CARcinogen Exposure database and expert-derived thresholds, while low-exposure industries (e.g., education, finance) received lower rates [1, 13].

**2.4 Methodology to calculate attributable burden in the GBD framework**

To quantify the disease burden attributable to a specific risk factor, the GBD study used a validated comparative risk assessment (CRA) framework [1]. In brief, the first step is quantifying the relative risks (RRs) of the health outcome (TBL cancer) as a function of exposure to the risk factor (occupational PAHs). This was done using a meta-regression in a “burden of proof” approach, which synthesizes data from systematic reviews. Second, exposure data are projected to the global scale from various sources using Bayesian statistical models, specifically spatiotemporal Gaussian process regression. Third, by integrating projected exposure levels, attributable burden could be calculated to quantify the proportional change in TBL cancer burden that would occur if occupational PAH exposure was reduced to the TMREL. Fourth, the GBD framework accounts for the joint effects of risk factors by assuming that RRs are multiplicative. For risk factors without mediating pathways, such as occupational PAH exposure and confounding factors (e.g., smoking and other occupational carcinogens), their independent contributions were calibrated to avoid overestimation of joint effects [1]. More detailed descriptions on the statistical methods, input data, and exposure-response functions are publicly accessible online (https://ghdx.healthdata.org/record/ihme-data/gbd-2021-burden-by-risk-1990-2021). Finally, all related estimates were recorded in the GBD 2021 result tools (https://vizhub.healthdata.org/gbd-results/).

*2. The APC analysis is interesting but complex. Could you provide more details on how the age, period, and cohort effects were separated, and what assumptions were made in this modeling?*

Response: Thank you for this valuable comment. We have added more details on this: In addition, to characterize the effects of age, cohort, and period on TBL cancer death rates, an age-period-cohort (APC) model was applied [14, 15]. This model helps separate the influence of the age effect (the age at which individuals are with higher risks), the generation or cohort effect (the impact of birth year on risks), and the period effect (the impact of time-specific changes on risks) [14, 15]. It is mathematically expressed as $Y_{ijk}=\alpha+f(A_{i})+g(P_{j})+h(C_{k})+\varepsilon_{ijk}$, where$Y_{ijk}$ represents the outcome, α is the intercept, $f(A_{i})$ captures the age effect, $g(P_{j})$ accounts for period effects, $h(C_{k})$ denotes cohort effects, and $\varepsilon_{ijk}$ is the error term. To estimate these effects, the APC model employs the intrinsic estimator method to deal with the collinearity in the cohort constraint equation and allows for robust estimation of independent effects while addressing the identifiability problem [14, 15]. **(Method Section)**

*3. You note higher EAPCs in females compared to males globally. What might explain this gender difference, given that occupational PAH exposure has traditionally been higher in male-dominated industries?*

Response: Thank you for this important comment. It is true that although we found males had higher ASDR and DALYs than females, while females presented more significant increasing trends (EAPC). There might be several explanations. In many regions over the past three decades, women’s participation in industries with PAH exposure might have grown [16, 17]. In addition, differences in background lung cancer trends and biological reactions by sex may also amplify the apparent increase in PAH-attributable burden among women [16-18]. We have added potential explanations in the **Discussion Section**:

Although males experienced higher ASDRs and DALYs attributable to occupational PAH exposure, females showed larger increasing trends indicated by higher EAPCs. This might be driven by several factors. First, over the past three decades, female workforce participation in sectors with incidental PAH exposure has increased in many countries [13, 16]. A previous pooled analysis of 14 case-control studies in Europe and Canada also reported higher risks of lung cancer for ever‑exposed women (OR=1.20) versus men (OR=1.08), despite lower median cumulative PAH exposure levels among women [16]. Experimental evidence also showed that women might be more susceptible to PAH-related toxicity due to biological differences. Studies have reported higher levels of oxidative stress biomarkers and genotoxic effects in exposed women than in men at similar exposure levels [17]. Furthermore, female lung tissue exhibits higher expression of CYP1A1 and greater accumulation of PAH–DNA adducts, suggesting enhanced metabolic activation of PAHs in women [19]. These biological susceptibilities may amplify the health impact of even modest occupational exposures, contributing to the more significant increasing trends observed in females. **(Discussion Section)**

*4.The inverted U-shaped relationship between SDI and TBL cancer burden is intriguing. Could you discuss potential mechanisms behind why middle SDI regions show the highest burden?*

Response: We agree and appreciate your comment. We have added related discussions according to your comments: In low to middle SDI regions, rapid industrialization particularly in sectors with high PAH exposure risks, such as manufacturing (e.g., aluminum production, coal tar distillation), construction, and informal waste management drives elevated occupational exposure [9, 10]. Meanwhile, inadequate protective measures such as poor ventilation and limited personal protective equipment may also aggravate related disease burden. This trend is particularly pronounced in regions such as East Asia, where accelerated industrialization in the late 20th century coincided with delayed adoption of workplace safety standards [11, 12]. The less apparent decline in females in high SDI regions also suggests potential gender-related disparities in occupational exposure patterns [12]. As regions transition to high SDI, advancements in occupational health policies, automation, and cleaner technologies reduce direct PAH exposure. Stricter regulatory frameworks combined with shifts toward service-oriented economies may also mitigate the TBL cancer burden attributed to occupational exposure to PAHs [6, 8]. **(Discussion Section)**

*5. The manuscript acknowledges uncertainties in GBD estimates. How might underreporting of occupational exposures in low/middle SDI regions affect your findings?*

Response: Thank you for raising this comment. The GBD framework utilizes models like spatiotemporal Gaussian process regression (ST-GPR) to pool this heterogeneous data. These models are crucial for controlling and adjusting for bias and for generating estimates in locations or time periods where direct data may be limited [1]. Nevertheless, it is true that one inherent limitation of the GBD study is the relatively poor data in lower SDI regions. We have added this as a limitation of our study: First, a key limitation of our study is that the GBD framework is subjected to relatively incomplete and heterogeneous data from LMICs. While the use of spatiotemporal Gaussian process regression helps mitigate data gaps by integrating multiple sources and adjusting for bias, substantial underreporting of occupational exposures and TBL cancer cases in LMICs may lead to underestimations of the true burden [1]. Our findings for lower SDI regions should therefore be interpreted as conservative estimates. **(Discussion Section)**

*6. Smoking is a major confounder for lung cancer. How was smoking controlled for in the attribution of TBL cancer to occupational PAH exposure?*

Response: Thank you for this important comment. Within the GBD comparative risk assessment framework, each risk factor is treated as an independent risk factor with its own exposure–response function. The exposure-response functions were derived from meta-analyses based on current evidence. When combining multiple risks, the GBD assumes multiplicative joint effects and applies a calibration step to prevent overestimation of total attributable fractions. Thus, the attributable burden to PAHs could characterize TBL cancer burden beyond that explained by smoking and other major risk factors [1]. We have added related content in the **Method Section**:

**2.4 Methodology to calculate attributable burden in the GBD framework**

To quantify the disease burden attributable to a specific risk factor, the GBD study used a validated comparative risk assessment (CRA) framework [1]. In brief, the first step is quantifying the relative risks (RRs) of the health outcome (TBL cancer) as a function of exposure to the risk factor (occupational PAHs). This was done using a meta-regression in a “burden of proof” approach, which synthesizes data from systematic reviews. Second, exposure data are projected to the global scale from various sources using Bayesian statistical models, specifically spatiotemporal Gaussian process regression. Third, by integrating projected exposure levels, attributable burden could be calculated to quantify the proportional change in TBL cancer burden that would occur if occupational PAH exposure was reduced to the TMREL. Fourth, the GBD framework accounts for the joint effects of risk factors by assuming that RRs are multiplicative. For risk factors without mediating pathways, such as occupational PAH exposure and confounding factors (e.g., smoking and other occupational carcinogens), their independent contributions were calibrated to avoid overestimation of joint effects [1]. More detailed descriptions on the statistical methods, input data, and exposure-response functions are publicly accessible online (https://ghdx.healthdata.org/record/ihme-data/gbd-2021-burden-by-risk-1990-2021). Finally, all related estimates were recorded in the GBD 2021 result tools (<https://vizhub.healthdata.org/gbd-results/>). **(Method Section)**

*7. Given your findings, what specific interventions would you recommend for low/middle SDI regions to reduce occupational PAH exposure?*

Response: Thank you for this valuable comment. We have related content in Discussion as: To reduce the global burden of TBL cancer associated with occupational PAH exposure, policymakers should prioritize interventions that align with the socio-economic and industrial contexts of regions at different SDI levels. Particularly, in low- and middle-SDI regions, where industrialization often outpaces occupational safety infrastructure, governments should enforce stricter workplace exposure limits for PAHs and the use of personal protective equipment. **(Discussion Section)**

*8. How might your results inform international occupational health standards for PAH exposure limits?*

Response: Thank you for this important comment. We have related content in Discussion as: Globally, harmonizing occupational safety standards through frameworks such as the ILO conventions and promoting technology transfer from high- to low-resource settings could mitigate disparities. Additionally, integrating gender-specific protections such as targeted safety protocols in female-dominated occupations would also be potentially helpful to mitigate related health burden. **(Discussion Section)**

*9. Have you considered analyzing specific high-risk occupations (e.g.,* *asphalt workers, coke oven workers) where PAH exposure is particularly high?*

Response: We appreciate this suggestion. We agree that focusing on occupations with exceptionally high PAH exposure would provide valuable insights. Unfortunately, the publicly available GBD occupational risk estimates are aggregated across broad industry classifications and do not allow disaggregation to individual job titles such as asphalt workers or coke oven workers. Therefore, we were unable to perform occupation-specific analyses. We have noted this in Methods, and have added this as a limitation.

In brief, occupational exposure to PAHs was quantified within the GBD framework using a multi-step process integrating economic activity classifications, occupation distributions, and exposure risk levels [13]. The input data were primarily sourced from the International Labor Organization (ILO). Occupational PAH exposure levels (high/low) were categorized based on the 17 International Standard Industrial Classification (ISIC) economic activities and 9 International Standard Classification of Occupations (ISCO) occupational categories. According to the GBD framework, the Theoretical Minimum Risk Exposure Level (TMREL) of occupational exposure to PAHs was set to zero (no thresholds). High-exposure industries (e.g., coal mining, asphalt production) were assigned elevated exposure rates using the CARcinogen Exposure database and expert-derived thresholds, while low-exposure industries (e.g., education, finance) received lower rates [1, 13]. **(Method Section)**

Fourth, the GBD framework aggregates occupational exposure across broad industry categories and does not allow further analysis by specific occupations. Consequently, our results could only reflect the average burden among all workers and do not capture the elevated risks faced by the heavily exposed jobs, such as asphalt workers or coke oven workers. **(Discussion Section)**

10. Were you able to examine dose-response relationships given the available data?

Response: Thank you for this question. We did not perform an independent dose-response analysis. Our study relied on the GBD framework, which used exposure-response functions publicly available (https://ghdx.healthdata.org/record/ihme-data/gbd-2021-burden-by-risk-1990-2021). We have made this clearer in the **Method Section:**

More detailed descriptions on the statistical methods, input data, and exposure-response functions are publicly accessible online (https://ghdx.healthdata.org/record/ihme-data/gbd-2021-burden-by-risk-1990-2021). Finally, all related estimates were recorded in the GBD 2021 result tools (<https://vizhub.healthdata.org/gbd-results/>). **(Method Section)**

References:

1.	GBD 2021 Risk Factors Collaborators: **Global burden and strength of evidence for 88 risk factors in 204 countries and 811 subnational locations, 1990-2021: a systematic analysis for the Global Burden of Disease Study 2021**. *Lancet (London, England)* 2024, **403**(10440):2162-2203.

2.	Mogaraju JK: **Machine learning strengthened prediction of tracheal, bronchus, and lung cancer deaths due to air pollution**. *Environmental science and pollution research international* 2023, **30**(45):100539-100551.

3.	Lu J, Zhao X, Gan S: **Global, regional and national burden of tracheal, bronchus, and lung cancer attributable to ambient particulate matter pollution from 1990 to 2021: an analysis of the global burden of disease study**. *BMC public health* 2025, **25**(1):108.

4.	Li H, Guo J, Liang H, Zhang T, Zhang J, Wei L, Shi D, Zhang J, Wang Z: **The Burden of Trachea, Bronchus, and Lung Cancer Attributable to Occupational Exposure From 1990 to 2019**. *Frontiers in public health* 2022, **10**:928937.

5.	Min L, Mao Y, Lai H: **Burden of silica-attributed pneumoconiosis and tracheal, bronchus & lung cancer for global and countries in the national program for the elimination of silicosis, 1990-2019: a comparative study**. *BMC public health* 2024, **24**(1):571.

6.	Louro H, Gomes BC, Saber AT, Iamiceli AL, Göen T, Jones K, Katsonouri A, Neophytou CM, Vogel U, Ventura C *et al*: **The Use of Human Biomonitoring to Assess Occupational Exposure to PAHs in Europe: A Comprehensive Review**. *Toxics* 2022, **10**(8).

7.	MacDonald RD, Thomas L, Rusk FC, Marques SD, McGuire D: **Occupational health and safety assessment of exposure to jet fuel combustion products in air medical transport**. *Prehospital emergency care* 2010, **14**(2):202-208.

8.	Jang TW, Kim Y, Won JU, Lee JS, Song J: **The standards for recognition of occupational cancers related with polycyclic aromatic hydrocarbons (PAHs) in Korea**. *Annals of occupational and environmental medicine* 2018, **30**:13.

9.	Sun Y, Kan Z, Zhang ZF, Song L, Jiang C, Wang J, Ma WL, Li YF, Wang L, Liu LY: **Association of occupational exposure to polycyclic aromatic hydrocarbons in workers with hypertension from a northeastern Chinese petrochemical industrial area**. *Environmental pollution (Barking, Essex : 1987)* 2023, **323**:121266.

10.	Borgulat J, Staszewski T: **Fate of PAHs in the vicinity of aluminum smelter**. *Environmental science and pollution research international* 2018, **25**(26):26103-26113.

11.	Rehman MYA, Taqi MM, Hussain I, Nasir J, Rizvi SHH, Syed JH: **Elevated exposure to polycyclic aromatic hydrocarbons (PAHs) may trigger cancers in Pakistan: an environmental, occupational, and genetic perspective**. *Environmental science and pollution research international* 2020, **27**(34):42405-42423.

12.	Yuan H, Wang Y, Duan H: **Risk of Lung Cancer and Occupational Exposure to Polycyclic Aromatic Hydrocarbons Among Workers Cohorts - Worldwide, 1969-2022**. *China CDC weekly* 2022, **4**(17):364-369.

13.	GBD 2016 Occupational Carcinogens Collaborators: **Global and regional burden of cancer in 2016 arising from occupational exposure to selected carcinogens: a systematic analysis for the Global Burden of Disease Study 2016**. *Occupational and environmental medicine* 2020, **77**(3):151-159.

14.	Yang Y, Fu WJ, Land KC: **A Methodological Comparison of Age-Period-Cohort Models: The Intrinsic Estimator and Conventional Generalized Linear Models**. *Sociological Methodology* 2004, **34**(1):75-110.

15.	Nasreen S, Wilk P, Mullowney T, Karp I: **Age, period, and cohort effects on asthma prevalence in Canadian adults, 1994-2011**. *Annals of epidemiology* 2020, **41**:49-55.

16.	Olsson A, Guha N, Bouaoun L, Kromhout H, Peters S, Siemiatycki J, Ho V, Gustavsson P, Boffetta P, Vermeulen R *et al*: **Occupational Exposure to Polycyclic Aromatic Hydrocarbons and Lung Cancer Risk: Results from a Pooled Analysis of Case-Control Studies (SYNERGY)**. *Cancer epidemiology, biomarkers & prevention : a publication of the American Association for Cancer Research, cosponsored by the American Society of Preventive Oncology* 2022, **31**(7):1433-1441.

17.	Guo H, Huang K, Zhang X, Zhang W, Guan L, Kuang D, Deng Q, Deng H, Zhang X, He M *et al*: **Women are more susceptible than men to oxidative stress and chromosome damage caused by polycyclic aromatic hydrocarbons exposure**. *Environmental and molecular mutagenesis* 2014, **55**(6):472-481.

18.	Uppstad H, Osnes GH, Cole KJ, Phillips DH, Haugen A, Mollerup S: **Sex differences in susceptibility to PAHs is an intrinsic property of human lung adenocarcinoma cells**. *Lung cancer (Amsterdam, Netherlands)* 2011, **71**(3):264-270.

19.	Mollerup S, Berge G, Baera R, Skaug V, Hewer A, Phillips DH, Stangeland L, Haugen A: **Sex differences in risk of lung cancer: Expression of genes in the PAH bioactivation pathway in relation to smoking and bulky DNA adducts**. *International journal of cancer* 2006, **119**(4):741-744.
