# Supplementary material for: Global burden and trends of tracheal, bronchus, and lung cancer attributed to occupational exposure to polycyclic aromatic hydrocarbons in regions with different sociodemographic index, 1990–2021
Source: PLoS One. 2026 Feb 12;21(2):e0342250. doi: 10.1371/journal.pone.0342250 (PMC12900364; doi:10.1371/journal.pone.0342250)
Supplement: Attachment — Submitted filename: 20160119Response letter.docx [file pone.0342250.s010.docx]

PONE-D-25-19840R1

Global burden and trends of tracheal, bronchus, and lung cancer attributed to occupational exposure to polycyclic aromatic hydrocarbons in regions with different sociodemographic index, 1990-2021

PLOS ONE

**Response letter**

Dear Dr. Igor Burstyn,

We appreciate your insightful feedback. We have carefully revised the manuscript in accordance with recommendations and believe that these comments have significantly enhanced the manuscript. A point-by-point response to the comments is attached along with the revised manuscript.

We appreciate your guidance and look forward to your response.

Sincerely,

Xiaowei Xie

Department of Thoracic Surgery, The First Hospital of Putian City, Putian, 351100, China

Email: xxw_biolab@163.com

***Journal Requirements:***

*If the reviewer comments include a recommendation to cite specific previously published works, please review and evaluate these publications to determine whether they are relevant and should be cited. There is no requirement to cite these works unless the editor has indicated otherwise.*

Response: Thank you for your remind. We have carefully reviewed the suggested publication to ensure appropriate citations.

***Comments to the Author*** *1. If the authors have adequately addressed your comments raised in a previous round of review and you feel that this manuscript is now acceptable for publication, you may indicate that here to bypass the “Comments to the Author” section, enter your conflict of interest statement in the “Confidential to Editor” section, and submit your "Accept" recommendation.*

Reviewer #3: All comments have been addressed

Reviewer #4: (No Response)

*2. Is the manuscript technically sound, and do the data support the conclusions?
The manuscript must describe a technically sound piece of scientific research with data that supports the conclusions. Experiments must have been conducted rigorously, with appropriate controls, replication, and sample sizes. The conclusions must be drawn appropriately based on the data presented.*

Reviewer #3: Partly

Reviewer #4: Yes

*3. Has the statistical analysis been performed appropriately and rigorously?*

Reviewer #3: Yes

Reviewer #4: Yes

*4. Have the authors made all data underlying the findings in their manuscript fully available?*
The PLOS Data policy requires authors to make all data underlying the findings described in their manuscript fully available without restriction, with rare exception (please refer to the Data Availability Statement in the manuscript PDF file). The data should be provided as part of the manuscript or its supporting information, or deposited to a public repository. For example, in addition to summary statistics, the data points behind means, medians and variance measures should be available. If there are restrictions on publicly sharing data—e.g. participant privacy or use of data from a third party—those must be specified.

Reviewer #3: Yes

Reviewer #4: Yes

*5. Is the manuscript presented in an intelligible fashion and written in standard English?*
PLOS ONE does not copyedit accepted manuscripts, so the language in submitted articles must be clear, correct, and unambiguous. Any typographical or grammatical errors should be corrected at revision, so please note any specific errors here.

Reviewer #3: Yes

Reviewer #4: Yes

*6. Review Comments to the Author*
Please use the space provided to explain your answers to the questions above. You may also include additional comments for the author, including concerns about dual publication, research ethics, or publication ethics. (Please upload your review as an attachment if it exceeds 20,000 characters)

**Reviewer #3:** 
*1. Over-reliance on GBD Modelling and Data Limitations:
While the authors have expanded the limitations section, the core issue remains that the study's findings are entirely dependent on the GBD's modelled estimates. The acknowledgement of incomplete and heterogeneous data from Low- and Middle-Income Countries (LMICs) is appropriate. However, the statement that findings for lower SDI regions are* *"conservative estimates" is speculative. The direction of bias (underestimation vs. overestimation) is difficult to ascertain due to competing factors: underreporting of occupational exposures and cancer cases versus potential over-attribution of TBL cancer to PAHs in the absence of robust confounder control at the* *individual level. This fundamental uncertainty should be more prominently and forcefully stated in the Abstract, Results, and Discussion, framing the entire interpretation of SDI disparities. A sensitivity analysis, though ideal, may not be feasible; therefore, the discussion of this limitation must be exceptionally strong.*
Response: We would like to thank you for your thoughtful considerations on the limitation of our manuscript. We agree with your comments and have expanded and added related sections.

Revisions:

Abstracts: “Nevertheless, given the inherent limitations of GBD estimation methods and data scarcity in LMICs, the observed disparities should be interpreted with caution and warrant further primary research.”

Results: In the first paragraph of the Discussion Section, after the overview of the results, we have added “Nevertheless, it is important to note that the comparisons across SDI regions are based on these modelled estimates, and the observed trends should be interpreted considering potential variability in data quality and completeness underlying the models, particularly in LMICs.”

Discussion: We have removed the “conservative estimates”, and have added related content: “First, a significant limitation of this study is its reliance on the modelled estimates of the GBD study. While the GBD employs robust methodologies to synthesize data and address gaps, the accuracy of its estimates is contingent on the quality and coverage of underlying source data. Substantial under-reporting of PAH exposures and TBL cancer diagnoses in many LMICs is a recognized issue, potentially leading to underestimation of the burden [1]. Meanwhile, the ecological nature of the analysis could not fully consider the confounders at the individual level (e.g., tobacco smoking, other occupational carcinogens), which means that the direction and magnitude of net bias are difficult to determine precisely [2]. Therefore, the observed disparities across SDI regions should be interpreted considering potential uncertainties.”

*2. Superficial Exploration of Gender Differences:
The explanation for higher Estimated Annual Percentage Change (EAPC) in females, while improved, leans heavily on biological susceptibility. This requires more critical balance. The authors should delve deeper into potential socio-occupational factors:*

*Segregation within industries: Are women increasingly entering specific roles within high-PAH industries that might have different exposure profiles (e.g., administrative roles in manufacturing vs. direct labor)?*

*Informal sector work: In many LMICs, women are disproportionately represented in informal waste management or small-scale industries where PAH exposure is unmeasured and unregulated. Could this contribute to the trend?*

*Interaction with other risk factors: The discussion should explicitly consider if trends in female smoking (which vary dramatically by region) could interact with or confound the observed PAH-attributable burden trends. The GBD's adjustment method is described, but its effectiveness in disentangling these correlated risks at the population level warrants a more cautious tone.*
Response: We agree with your comments and thank you for this remind. We have improved this section as suggested.

Revision: “Although males experienced higher ASDRs and DALYs attributable to occupational PAH exposure, females showed larger increasing trends indicated by higher EAPCs. This observed pattern may be driven by a confluence of socioeconomic, occupational, and biological factors. First, shifting occupational demographics play a role. While female workforce participation in formal sectors with potential PAH exposure has increased, gender-based occupational segregation often places women in different roles within these industries (e.g., administrative or support functions versus direct production labor) [3, 4]. More critically, in many LMICs, women are disproportionately represented in the informal economy, including waste picking, small-scale food processing using solid fuels, and home-based manufacturing, where high-intensity PAH exposures are common and outside regulatory frameworks [5]. This unregulated exposure likely contributes substantially to the rising trend. Second, the influence of correlated risk factors, particularly active smoking, warrants consideration [1, 6, 7]. As highlighted by a study among the ten most populous countries, while tobacco-associated lung cancer mortality rates have declined among males in most countries, they have increased among females from 1990 to 2019 [7]. Notably, historical smoking trends have differed by sex, with female smoking prevalence peaking later than male prevalence in many regions [6, 8]. Consequently, recent trends in female lung cancer burden likely reflect the combined effects of both risk factors. Although the GBD framework adjusts for smoking independently [1], residual confounding or interaction between smoking and occupational PAHs at the population level may influence the observed sex-specific trends. Third, biological susceptibility may amplify the trends. A previous pooled analysis of 14 case-control studies in Europe and Canada also reported higher risks of lung cancer for ever‑exposed women (OR=1.20) versus men (OR=1.08), despite lower median cumulative PAH exposure levels among women [3]. Experimental evidence also showed that women might be more susceptible to PAH-related toxicity due to biological differences. Studies have reported higher levels of oxidative stress biomarkers and genotoxic effects in exposed women than in men at similar exposure levels [9]. Furthermore, female lung tissue exhibits higher expression of CYP1A1 and greater accumulation of PAH–DNA adducts, suggesting enhanced metabolic activation of PAHs in women [10]. These biological susceptibilities may amplify the health impact of even modest occupational exposures, contributing to the more significant increasing trends observed in females.”

*3. Mechanistic Explanation of the SDI-Burden Relationship:
The added discussion on the inverted U-shaped curve is helpful but still somewhat descriptive. To strengthen the mechanistic insight:*

*Link to Economic Transition Theories: Frame the findings within established theories of epidemiological or risk transition. The peak burden in middle-SDI regions mirrors patterns seen with other occupational and environmental hazards, where industrialization outpaces regulatory capacity and health infrastructure.*

*Latency and Temporal Misalignment: Emphasize that the current burden in high-SDI regions reflects historical exposures from decades past (when their SDI was middle-range). Conversely, the current high burden in middle-SDI regions is a "real-time" consequence of present-day exposures. This temporal disconnect between exposure (past/present) and outcome (present/future) is crucial for interpreting the APC results and forecasting future burdens. The discussion should more explicitly connect the period/cohort effects to these historical industrialization waves.*
Response: Fully agree. We have added this content in revised manuscript.

Revision: “Our analysis identified an inverted U-shaped relationship between SDI and TBL cancer burden, which highlights complex interactions between socioeconomic development and TBL cancer burden associated with occupational exposures to PAHs. The observed inverted U-shaped relationship between SDI and PAH-attributable TBL cancer burden aligns with the framework of the epidemiological transition of occupational risk. In this paradigm, the population-level burden peaks when the pace of industrialization and resultant exposure outstrip the development of protective regulatory frameworks and healthcare capacity. This is evident in low- to middle-SDI regions, where expansion in high-exposure sectors (e.g., manufacturing, construction, informal industry) has driven increased occupational PAH exposure [11, 12], while safeguards such as ventilation controls and personal protective equipment remain inadequate. This trend is particularly pronounced in regions such as East Asia, where accelerated industrialization in the late 20th century coincided with the delayed adoption of workplace safety standards [13, 14]. The high burden observed today in middle-SDI regions is largely attributable to ongoing, intensive exposures from current industrial activities. In contrast, the contemporary burden in high-SDI regions primarily stems from exposures accumulated in the past, when these regions were at a similar developmental stage. The declining burden trends now seen in high-SDI settings represent the delayed benefit of occupational health policies, technological improvements, and economic shifts implemented in prior decades [15, 16].

The APC analyses further provided additional insights into the temporal dynamics of TBL cancer burden attributed to occupational PAH exposure. It should be noted that the interpretation of cohort patterns is inherently limited by the 30-year study observation window and the multi-decadal latency of TBL cancer, meaning the observed cohort effect integrates exposure experiences over a much longer historical timeframe. Particularly, the strong and increasing period effect in low- and middle-SDI regions quantifies the persistent risk from present-day industrial exposure [16-18]. In high-SDI regions, a more modest period effect coupled with elevated risk in older cohorts captures the enduring impact of past exposure regimes [19, 20]. Meanwhile, the declining cohort effect observed globally suggests that preventive measures may be reducing risk for more recent generations [20]. Together, these APC findings demonstrate that the inverted U-shaped curve is not merely a cross-sectional disparity but a dynamic signature of industrialization waves, exposure histories, and the delayed translation of TBL cancer burden attributed to occupational PAH exposure.”

*4. Policy Recommendations Lack Specificity and Feasibility Analysis:
The policy section remains generic. To enhance impact, recommendations should be more targeted and actionable:*

*Beyond PPE and Limits: While important, recommending "stricter exposure limits and PPE" in low-resource settings is often infeasible without parallel investments in enforcement, monitoring, and worker education. Suggest concrete, incremental steps (e.g., prioritizing exposure control in 2-3 key industries, promoting simple ventilation improvements, developing low-cost exposure biomarkers for surveillance).*

*Leveraging Existing Frameworks: Mentioning ILO conventions is good. Specify which conventions (e.g., C139, C155, C170) are most relevant and propose a tangible pathway for their adoption and monitoring in target regions.*

*Case Examples: Briefly reference a successful intervention from a specific country (e.g., reduction in PAH exposures in a particular industry in a high-SDI setting) and discuss its potential for adaptation.*

*Research-to-Policy Pipeline: Recommend establishing linked occupational cancer registries in sentinel middle-SDI industrial zones to improve data quality and directly inform local policy, moving beyond reliance on global models.*
Response: We appreciate your comments. According to your comments, we have expanded related content.

Revision: “To reduce the global burden of TBL cancer attributable to occupational PAH exposure, policymakers should prioritize interventions that align with the socio-economic and industrial contexts of regions at different SDI levels. Particularly, in low- and middle-SDI regions, where industrialization often outpaces occupational safety infrastructure, governments should enforce stricter workplace exposure limits for PAHs and the use of personal protective equipment. Efforts should prioritize exposure controls within high-risk industries (e.g., small-scale manufacturing, brick kilns, and informal waste recycling) and practical interventions (e.g., improving natural ventilation, providing access to affordable respiratory protection, and delivering basic occupational safety training) [19]. Globally, harmonizing standards should focus on promoting the ratification and implementation of relevant ILO conventions (https://www.ilo.org/), specifically the Occupational Safety and Health Convention (No. 155) and the Chemicals Convention (No. 170), supported by technical cooperation for monitoring and enforcement. Successful PAH exposure reduction strategies from high-SDI settings, such as the use of enclosed processes and local exhaust ventilation in coke oven operations, offer transferable models that could be adapted to local industrial conditions [19, 21]. Furthermore, strengthening local data systems is essential to move beyond reliance on modelled estimates. Establishing linked occupational cancer and exposure registries in sentinel industrial zones of middle-SDI countries would generate locally relevant evidence to directly guide and evaluate policy. Finally, given the rising disease trends among females, integrating gender-specific protections such as targeted safety protocols in female-dominated occupations would also be potentially helpful to mitigate related health burden.”

**Minor:**
*1. APC Analysis Interpretation: The interpretation of cohort effects ("**recent generations might have lower death rates") is challenging given the 30-year study window and the long latency of TBL cancer. The observed cohort effect likely reflects exposures from the mid-20th century onwards. This complexity should be acknowledged to avoid oversimplification.*

Response: Thank you for this remind. We have added related discussions on this.

Revision: “The APC analyses further provided additional insights into the temporal dynamics of TBL cancer burden attributed to occupational PAH exposure. It should be noted that the interpretation of cohort patterns is inherently limited by the 30-year study observation window and the multi-decadal latency of TBL cancer, meaning the observed cohort effect integrates exposure experiences over a much longer historical timeframe. Particularly, the strong and increasing period effect in low- and middle-SDI regions quantifies the persistent risk from present-day industrial exposure [16-18]. In high-SDI regions, a more modest period effect coupled with elevated risk in older cohorts captures the enduring impact of past exposure regimes [19, 20]. Meanwhile, the declining cohort effect observed globally suggests that preventive measures may be reducing risk for more recent generations [20]. Together, these APC findings demonstrate that the inverted U-shaped curve is not merely a cross-sectional disparity but a dynamic signature of industrialization waves, exposure histories, and the delayed translation of TBL cancer burden attributed to occupational PAH exposure.”

*2. Figure and Table Presentation:
Table 1: The table is dense. Consider creating a separate, simplified summary table for the main global and SDI-region results in the main text and moving the full sex-stratified table to the supplement.*

*Figure 1 (Maps): Ensure the color scales are perfectly intuitive and include a clear note in the caption that the maps depict EAPC, not absolute burden.*
Response:

(1) Thank you for your suggestion. We have separated the results by sex into S1 Table in the Appendix.

2) Thank you for your remind. We have added the “EAPC” in the title of the figures to improve clarity.

*3. Language and Flow: The manuscript is generally well-written. A final careful edit for concise phrasing and to avoid minor repetitions (e.g., the GBD methodology is described in very similar terms in multiple sections) would enhance readability.*

Response: Thank you for this comment. We have performed a thorough edit of the entire manuscript to improve readability.

***Reviewer #4:***

*Thank you for the opportunity to review your work and I hope you find my comments of assistance. The manuscript addresses an important topic TBL cancer which has an increasing incidence, The attribution to preventable causes such as work is important.*
Response: We would like to thank you for your thoughtful considerations and comments for improving our manuscript.

*The striking finding for me is the sex differences which show a change in the direction of the usually expected ratios for occupational diseases. These changes in EAPC appear to be marked by country. Noting that the GBD methods for confounders were applied to what extent do the authors feel smoking rates in females may be contributory in comparison to those of males? The paper Evolving trends in lung cancer risk factors in the ten most populous countries: an analysis of data from the 2019 Global Burden of Disease Study Jani, Chinmay T. et al. ClinicalMedicine, Volume 79, 103033 may be helpful although it only examines a limited number of countries. However it is noted, "Among males, tobacco-associated ASMR fell over time across all countries, excluding China, Indonesia, and Pakistan. Among females, the reverse pattern was observed with overall increasing rates of tobacco-associated ASMR in most countries, with only rates in the USA and Mexico falling between 1990 and 2019".*

Response: Thank you for these comments. We agree that divergent historical trends in smoking prevalence between sexes are an important contextual factor that warrants careful consideration when interpreting the observed sex-specific trends in PAH-attributable burden. While the GBD framework statistically adjusts for smoking as an independent risk factor to estimate the burden attributable to occupational PAHs, the strong ecological correlation and overlapping secular trends between smoking and occupational PAH exposures at the population level mean that residual confounding or interaction could influence the estimated temporal trends. We have expanded the Discussion to incorporate this point.

Revision: “Second, the influence of correlated risk factors, particularly active smoking, warrants consideration [1, 6, 7]. As highlighted by a study among the ten most populous countries, while tobacco-associated lung cancer mortality rates have declined among males in most countries, they have increased among females from 1990 to 2019 [7]. Notably, historical smoking trends have differed by sex, with female smoking prevalence peaking later than male prevalence in many regions [6, 8]. Consequently, recent trends in female lung cancer burden likely reflect the combined effects of both risk factors. Although the GBD framework adjusts for smoking independently [1], residual confounding or interaction between smoking and occupational PAHs at the population level may influence the observed sex-specific trends.”

*In addition to the above to what extent is proportional mortality playing a role in survival to die of TBL?*

Response: Thank you for this insightful methodological point. Changes in mortality from other major causes (e.g., smoking) can alter the size and age-structure of the population at risk for lung cancer over time. This, in turn, could affect both the absolute number and the proportional contribution of deaths attributable to occupational PAHs. While the GBD framework estimates absolute attributable burden, ecological trend analyses do not explicitly isolate this effect. As our analyses only included one specific risk factor, we were not able to evaluate this. We have added this as a limitation.

Revision: “Sixth, the interpretation of long-term trends in attributable burden can be influenced by changes in other competing risk factors over time. Shifts in the other major risk factors may alter the underlying population at risk for lung cancer, which could affect trends in attributable burden estimates independent of changes in lung cancer burden attributable to occupational PAH exposure.”

*L169 "While occupational exposure primarily occurs during working-age years (typically 15–75 years), the disease burden manifests later in life due to the long latency period of TBL cancer." Figure 3 demonstrates that actually the majority of deaths occur <75 years age**. It is perhaps more useful to state* *the typical latency of TBL cancer.*

Response: We agree that stating a more specific timeframe related to disease progression is more informative. For occupational PAH-related lung cancer, the induction-latency period (from first exposure to cancer diagnosis) is typically prolonged, often 10 years or more [22, 23]. Consequently, although exposure occurs during working ages (15~75 years), diagnosis and subsequent mortality are shifted to later ages. Therefore, to comprehensively capture the mortality burden arising from earlier exposures, our analysis included disease burden from 25 to >95 years.

Revision: “Occupational exposure to PAHs occurs primarily during working-age years (15-75 years). However, due to a prolonged induction-latency period of typically over 10 years from initial exposure to cancer diagnosis [22, 23], the attributable mortality burden is observed at older ages.”

*L49 "Increasing studies have reported disparities in incidence...", suggest "An increasing number of studies have reported disparities in incidence ..."*

Response: Thank you for your specific comments. Revised as suggested.

*L55 "**PAHs are known carcinogens to increase the risk of TBL cancer" suggest "**PAHs are carcinogens known to increase the risk of TBL cancer"*

Response: Revised as suggested.

*L64 "PAH exposure, a significant while overlooked risk factor" suggest "PAH exposure, a significant although overlooked risk factor"*

Response: Revised as suggested.

*L446 "subjected to relatively incomplete and heterogeneous data from LMICs." suggest "**subject to relatively incomplete and heterogeneous data from LMICs."*

Response: We have revised the content as “Substantial under-reporting of PAH exposures and TBL cancer diagnoses in many LMICs is a recognized issue, potentially leading to underestimation of the burden”, also given the comments from Reviewer 3.

*Figure 5 is interesting but particularly difficult to distinguish the markers for "Both" and "Female" even when I zoom*

Response: Thank you for this comment. It is true that there are many regions and there are some overlaps. We have improved the resolution of the symbols and the figure. We also uploaded the original Figure files in the submission system.

References:

1.	GBD 2021 Risk Factors Collaborators: **Global burden and strength of evidence for 88 risk factors in 204 countries and 811 subnational locations, 1990-2021: a systematic analysis for the Global Burden of Disease Study 2021**. *Lancet* 2024, **403**(10440):2162-2203.

2.	Afshar N, English DR, Milne RL: **Factors Explaining Socio-Economic Inequalities in Cancer Survival: A Systematic Review**. *Cancer Control* 2021, **28**:10732748211011956.

3.	Olsson A, Guha N, Bouaoun L, Kromhout H, Peters S, Siemiatycki J *et al*: **Occupational Exposure to Polycyclic Aromatic Hydrocarbons and Lung Cancer Risk: Results from a Pooled Analysis of Case-Control Studies (SYNERGY)**. *Cancer Epidemiol Biomarkers Prev* 2022, **31**(7):1433-1441.

4.	GBD 2016 Occupational Carcinogens Collaborators: **Global and regional burden of cancer in 2016 arising from occupational exposure to selected carcinogens: a systematic analysis for the Global Burden of Disease Study 2016**. *Occup Environ Med* 2020, **77**(3):151-159.

5.	Badal S, Holland K, Foster M, Le AB: **Occupational Safety and Health Risks of Informal Waste Workers in Nepal: A Mapping Review**. *Saf Health Work* 2025, **16**(3):325-332.

6.	Olsson AC, Fevotte J, Fletcher T, Cassidy A, t Mannetje A, Zaridze D *et al*: **Occupational exposure to polycyclic aromatic hydrocarbons and lung cancer risk: a multicenter study in Europe**. *Occup Environ Med* 2010, **67**(2):98-103.

7.	Jani CT, Kareff SA, Morgenstern-Kaplan D, Salazar AS, Hanbury G, Salciccioli JD *et al*: **Evolving trends in lung cancer risk factors in the ten most populous countries: an analysis of data from the 2019 Global Burden of Disease Study**. *EClinicalMedicine* 2025, **79**:103033.

8.	Di Novi C, Marenzi A: **The smoking epidemic across generations, genders, and educational groups: A matter of diffusion of innovations**. *Economics & Human Biology* 2019, **33**:155-168.

9.	Guo H, Huang K, Zhang X, Zhang W, Guan L, Kuang D *et al*: **Women are more susceptible than men to oxidative stress and chromosome damage caused by polycyclic aromatic hydrocarbons exposure**. *Environ Mol Mutagen* 2014, **55**(6):472-481.

10.	Mollerup S, Berge G, Baera R, Skaug V, Hewer A, Phillips DH *et al*: **Sex differences in risk of lung cancer: Expression of genes in the PAH bioactivation pathway in relation to smoking and bulky DNA adducts**. *Int J Cancer* 2006, **119**(4):741-744.

11.	Sun Y, Kan Z, Zhang ZF, Song L, Jiang C, Wang J *et al*: **Association of occupational exposure to polycyclic aromatic hydrocarbons in workers with hypertension from a northeastern Chinese petrochemical industrial area**. *Environ Pollut* 2023, **323**:121266.

12.	Borgulat J, Staszewski T: **Fate of PAHs in the vicinity of aluminum smelter**. *Environ Sci Pollut Res Int* 2018, **25**(26):26103-26113.

13.	Rehman MYA, Taqi MM, Hussain I, Nasir J, Rizvi SHH, Syed JH: **Elevated exposure to polycyclic aromatic hydrocarbons (PAHs) may trigger cancers in Pakistan: an environmental, occupational, and genetic perspective**. *Environ Sci Pollut Res Int* 2020, **27**(34):42405-42423.

14.	Yuan H, Wang Y, Duan H: **Risk of Lung Cancer and Occupational Exposure to Polycyclic Aromatic Hydrocarbons Among Workers Cohorts - Worldwide, 1969-2022**. *China CDC Wkly* 2022, **4**(17):364-369.

15.	Louro H, Gomes BC, Saber AT, Iamiceli AL, Göen T, Jones K *et al*: **The Use of Human Biomonitoring to Assess Occupational Exposure to PAHs in Europe: A Comprehensive Review**. *Toxics* 2022, **10**(8).

16.	Jang TW, Kim Y, Won JU, Lee JS, Song J: **The standards for recognition of occupational cancers related with polycyclic aromatic hydrocarbons (PAHs) in Korea**. *Ann Occup Environ Med* 2018, **30**:13.

17.	MacDonald RD, Thomas L, Rusk FC, Marques SD, McGuire D: **Occupational health and safety assessment of exposure to jet fuel combustion products in air medical transport**. *Prehosp Emerg Care* 2010, **14**(2):202-208.

18.	Wang W, Xu J, Qu X, Lin D, Yang K: **Current and Future Trends of Low and High Molecular Weight Polycyclic Aromatic Hydrocarbons in Surface Water and Sediments of China: Insights from Their Long-Term Relationships between Concentrations and Emissions**. *Environ Sci Technol* 2022, **56**(6):3397-3406.

19.	Driscoll TR, Carey RN, Peters S, Glass DC, Benke G, Reid A *et al*: **The Australian Work Exposures Study: Occupational Exposure to Polycyclic Aromatic Hydrocarbons**. *Ann Occup Hyg* 2016, **60**(1):124-131.

20.	Bottai M, Selander J, Pershagen G, Gustavsson P: **Age at occupational exposure to combustion products and lung cancer risk among men in Stockholm, Sweden**. *Int Arch Occup Environ Health* 2016, **89**(2):271-275.

21.	Vimercati L, Bisceglia L, Cavone D, Caputi A, De Maria L, Delfino MC *et al*: **Environmental Monitoring of PAHs Exposure, Biomarkers and Vital Status in Coke Oven Workers**. *Int J Environ Res Public Health* 2020, **17**(7).

22.	Bilello KS, Murin S, Matthay RA: **Epidemiology, etiology, and prevention of lung cancer**. *Clin Chest Med* 2002, **23**(1):1-25.

23.	Miller BG, Doust E, Cherrie JW, Hurley JF: **Lung cancer mortality and exposure to polycyclic aromatic hydrocarbons in British coke oven workers**. *BMC Public Health* 2013, **13**:962.
